# Sample Preparation and Liquid Chromatography-Tandem Mass Spectrometry for Multiple Steroids in Mammalian and Avian Circulation

**DOI:** 10.1371/journal.pone.0032496

**Published:** 2012-02-27

**Authors:** Lee Koren, Ella S. M. Ng, Kiran K. Soma, Katherine E. Wynne-Edwards

**Affiliations:** 1 Department of Comparative Biology & Experimental Medicine, Faculty of Veterinary Medicine, and Hotchkiss Brain Institute, University of Calgary, Calgary, Alberta, Canada; 2 Department of Psychology, University of British Columbia, Vancouver, British Columbia, Canada; Clermont Université, France

## Abstract

Blood samples from wild mammals and birds are often limited in volume, allowing researchers to quantify only one or two steroids from a single sample by immunoassays. In addition, wildlife serum or plasma samples are often lipemic, necessitating stringent sample preparation. Here, we validated sample preparation for simultaneous liquid chromatography – tandem mass spectrometry (LC-MS/MS) quantitation of cortisol, corticosterone, 11-deoxycortisol, dehydroepiandrosterone (DHEA), 17β-estradiol, progesterone, 17α-hydroxyprogesterone and testosterone from diverse mammalian (7 species) and avian (5 species) samples. Using 100 µL of serum or plasma, we quantified (signal-to-noise (S/N) ratio ≥10) 4–7 steroids depending on the species and sample, without derivatization. Steroids were extracted from serum or plasma using automated solid-phase extraction where samples were loaded onto C18 columns, washed with water and hexane, and then eluted with ethyl acetate. Quantitation by LC-MS/MS was done in positive ion, multiple reaction-monitoring (MRM) mode with an atmospheric pressure chemical ionization (APCI) source and heated nebulizer (500°C). Deuterated steroids served as internal standards and run time was 15 minutes. Extraction recoveries were 87–101% for the 8 analytes, and all intra- and inter-run CVs were ≤8.25%. This quantitation method yields good recoveries with variable lipid-content samples, avoids antibody cross-reactivity issues, and delivers results for multiple steroids. Thus, this method can enrich datasets by providing simultaneous quantitation of multiple steroids, and allow researchers to reimagine the hypotheses that could be tested with their volume-limited, lipemic, wildlife samples.

## Introduction

Immunoassays are widely used in clinical and comparative studies to measure circulating steroids [1]. Commercial antibody-based ELISA and RIA kits are readily available and relatively straightforward to use when measuring a single steroid. However, only one hormone can be measured from a sample, unless chromatography is employed to separate the steroids or the sample is split. Separate kits must then be used to quantify each steroid. Another disadvantage inherent to immunoassays is cross-reactivity of the antibody [2], [3]. Since steroids have similar structures, antibody cross-reactivity is high for some ELISA and RIA kits. This is especially problematic when measuring molecules with similar structures, such as multiple estrogens or glucocorticoids [4].

Liquid chromatography coupled with tandem mass spectrometry (LC-MS/MS) represents a good choice for the quantitation of steroids at wide-ranging concentrations. This technique avoids the issue of antibody cross-reactivity [5], [6], [7], [8]. Another advantage of LC-MS/MS is its ability to analyze multiple steroids simultaneously. Since sample volume is often limited when working with wild animals, it is invaluable to extract as much information as possible from every sample. With appropriate sample preparation, LC-MS/MS allows multiple steroids to be quantitated from a single sample [9], [10], [11], [12]. Lastly, LC-MS/MS can be automated, which allows high throughput.

In LC-MS/MS, sample preparation is especially important in order to maximize sensitivity and precision. Lipids can interfere with steroid measurement and are abundant in diverse biological matrices, such as serum from mammals preparing for hibernation or plasma from birds laying eggs. Traditionally, organic solvents have been used to extract steroids from biological matrices, but solid-phase extraction (SPE) gives superior results in terms of recovery and removal of interfering compounds [13], [14].

In clinical settings, multiple steroids have been measured in human serum using LC-MS/MS (e.g., [9]). The purpose of this study was to establish a core method that could be applied to a diverse array of wildlife blood samples in a research setting. Following the approach used for human serum, the expectation was that appropriate sample preparation would allow simultaneous quantitation of a panel of 8 steroid hormones including cortisol, corticosterone, 11-deoxycortisol, dehydroepiandrosterone (DHEA), 17β-estradiol (E_2_), progesterone (P_4_), 17α-hydroxyprogesterone (17-OH P) and testosterone (T_5_) in mammalian serum and avian plasma. The method was specifically developed using samples pooled across multiple individuals to maximize inclusion of potentially interfering matrix compounds and to represent an ‘average’ sample. The focus was on developing a sample preparation and analytic method that was robust across species with different matrix challenges, for serum or plasma samples, and that was sensitive enough across multiple steroids to have broad applicability for mammalian and avian wildlife samples.

## Materials and Methods

### Ethics Statement

No animals were sampled specifically for this research. All samples were secondary use of samples collected under animal use protocols overseen by Institutional Animal Care Committees and the Canadian Council on Animal Care. Thus, no animal use certification was required.

### Chemicals and reagents

Cortisol, corticosterone, dehydroepiandrosterone (DHEA), 17β-estradiol (E_2_) and testosterone (T_5_) were obtained from Sigma-Aldrich (St. Louis, MO, USA). 11-deoxycortisol, progesterone (P_4_) and 17α-hydroxyprogesterone (17-OH P) were from Steraloids Inc. (Newport, RI, USA). Deuterium labeled internal standards including cortisol–9,11,12,12-d4 (cortisol-*d*_4_), corticosterone-2,2,4,6,6,17α,21,21-*d*_8_ (corticosterone-*d*_8_), dehydroepiandrosterone-16,16-*d_2_* (DHEA-*d*_2_), 17β-estradiol-2,4,16,16-*d*_4_ (estradiol-*d*_4_), testosterone-1,2-d2 (testosterone-*d*_2_), 11-deoxycortisol-21-21-*d*_2_ (11-deoxycortisol-*d*_2_), progesterone-2,2,4,6,6,17α,21,21,21-d9 (progesterone-*d*_9_), and 4-pregnen-17α-ol-3,20-dione-2,2,4,6,6,21,21,21-d8 (17α-hydroxyprogesterone-*d*_8_) were purchased from C/D/N Isotopes Inc. (Pointe-Claire, QC, Canada). Optima-grade ethyl acetate, hexane, methanol and water were obtained from Fisher Scientific (Fair Lawn, NJ, USA). Bond Elut® C18 (100 mg, 1 mL endcapped) solid-phase extraction (SPE) cartridges were from Agilent Technologies (Santa Clara, CA, USA).

### Preparation of calibrators, internal standards and quality controls

Defibrinated and 4× charcoal stripped human serum (BioChemed Services, Winchester, VA) was used to prepare the calibration curves for quantitation of all serum and plasma samples in this paper.

Stock solutions of each steroid and internal standard (IS) were prepared separately in methanol at 1.0 mg/mL. The highest calibrator containing a mixture of 8 steroids was prepared by mixing appropriate volumes of each individual steroid stock solution and diluted with stripped human serum to obtain a final concentration of 1000 ng/mL cortisol, 10 ng/mL corticosterone, 5 ng/mL 11-deoxycortisol, and 10 ng/mL DHEA and 20 ng/mL each of E_2_, P_4_, 17-OH P and T_5_. An eight-point calibration curve (7 calibrators and a blank) was prepared by diluting the highest calibrator with stripped human serum. The concentrations of each calibrator are listed in Table 1.

**Table 1:** Concentrations of the eight calibrators (ng/mL) prepared with defibrinated and charcoal stripped human serum.

Calibrator	Cortisol	Corticosterone	11-deoxycortisol	DHEA	17β-Estradiol	Progesterone	17-OH Progesterone	Testosterone
1	0	0	0	0	0	0	0	0
2	5	0.05	0.025	0.1	0.1	0.1	0.1	0.1
3	10	0.1	0.05	0.2	0.2	0.2	0.2	0.2
4	25	0.25	0.125	0.5	0.5	0.5	0.5	0.5
5	50	0.5	0.25	1	1	1	1	1
6	250	2.5	1.25	5	5	5	5	5
7	500	5	2.5	10	10	10	10	10
8	1000*	10	5	20	20	20	20	20

* This calibrator was omitted because the concentration saturated the detector.

A mixture of eight internal standards was prepared by diluting each individual stock I.S. with methanol to obtain a final concentration of 20 ng/mL cortisol-*d*_4_, 10 ng/mL corticosterone-*d*_8_, 5 ng/mL DHEA-*d*_2_, 10 ng/mL 11-deoxycortisol-*d*_2_, 5 ng/mL estradiol-*d*_4_, 5 ng/mL progesterone-*d*_9_, 10 ng/mL 17α-hydroxyprogesterone-*d*_8_, and 10 ng/mL testosterone-*d*_2_. In-house quality controls (QC) were prepared at concentrations of 250 ng/mL cortisol, 2.5 ng/mL corticosterone, 5 ng/mL DHEA, 1.25 ng/mL 11-deoxycortisol, 5 ng/mL E_2_, 5 ng/mL P_4_, 5 ng/mL 17-OH P and 5 ng/mL T_5_ using stripped human serum.

### Sample preparation

As our focus was on optimization of the method, not assessment of species-typical concentrations within samples, all analysis was conducted using pooled serum samples. Each of the seven mammalian serum pools was created from at least 4 individuals, including 2 adult males and 2 adult non-pregnant females. Pooling, therefore, reduced testosterone concentration by dilution with female serum and reduced estradiol concentration by the inclusion of male serum. As such, challenges for quantitation sensitivity were deliberately increased for the sex steroids. In addition, this pooling was expected to enhance inclusion of potential interfering compounds from the matrix, while providing a large enough pool to assess alternate sample preparation and LC-MS/MS methods

Bactrian camel (*Camelus bactrianus*), moose (*Alces alces*), Siberian tiger (*Panthera tigris altaica*), Sri Lankan elephant (*Elephas maximus maximus*) and Przewalski's wild horse (p-horse; *Equus caballus przewalskii*) samples were obtained from the Calgary Zoo's serum bank. These species were chosen because their reproduction is recorded but not manipulated. Captive reindeer (*Rangifer tarandus*) serum samples were obtained in November 2009 following the rut, from the University of Calgary, and wild brown bear (*Ursus arctos*) serum samples were obtained in April 2010, before the mating season, from the Scandinavian Brown Bear Project.

In addition, we obtained avian plasma from captive birds (European starlings - *Sturnus vulgaris* and adult zebra finches - *Taeniopygia guttata*) at Simon Fraser University and the University of British Columbia respectively, as well as wild birds (black-browed albatross - *Thalassarche melanophrys*, house sparrows - *Passer domesticus*, and song sparrows - *Melospiza melodia*) from studies at the University of Calgary and University of British Columbia. For each species, pools were created across multiple individuals. However, where possible, pools were separated by sex to enhance the potential for detection of sex steroids. The starling and albatross pools each contained 6 females. The zebra finch pools contained breeding males (N = 7) and breeding females (N = 6). The single house sparrow pool contained 10 breeding males and 7 females. Song sparrow pools were separated into breeding males (N = 6) and non-breeding males (N = 5).

100 µL of serum or plasma samples, calibrators and quality controls were spiked with 20 µL of internal standards (final concentrations specified above) followed by 400 µL of water. Thus, calibration curves went through identical sample preparation and were derived in each analytical run. Sample processing was performed via an automated SPE system from Gilson Inc. (GX-274 ASPEC™, Gilson, Middleton, WI). Briefly, each sample was applied to a 1 mL Bond Elut® C18 SPE cartridge previously conditioned with methanol and water. The sample loading rate was 0.1 mL/min, and the samples were washed with 1 mL of water followed by 1 mL of hexane at a flow rate of 1 mL/min. The SPE cartridges were then dried for approximately 2 min and the 8 steroid analytes were eluted with 1 mL of ethyl acetate at a flow rate of 0.1 mL/min. Solvents were evaporated to dryness under a stream of high purity nitrogen at 45°C using a sample concentrator (Techne Inc., Burlington, NJ). The dry extracts were reconstituted in 100 µL of 50∶50 methanol/water, and 40 µL was injected into the LC-MS/MS.

### LC-MS/MS conditions

Following extraction, 40 µL of the reconstituted sample was injected into a 100×3.00 mm, 2.6 µm Kinetex® C18 HPLC column (Phenomenex, Torrance, CA) using an Agilent 1200 SL LC system with a thermostated autosampler set at 4°C (Agilent Technologies, Santa Clara, CA). The chromatographic separation was performed by a gradient elution (15 min) at a flow rate of 0.55 mL/min using water (mobile phase A) and methanol (mobile phase B). The LC system was coupled to an AB SCIEX QTRAP® 5500 tandem mass spectrometer (AB SCIEX, Concord, ON, Canada) fitted with an atmospheric pressure chemical ionization (APCI) source. The nebulizer current was set at 5 µA with a source temperature of 500°C. Nitrogen was produced by a high purity nitrogen generator (Parker Balston, MA, USA) and was utilized as the curtain, drying and collision gases. The elution gradient was held at 10% B for the first 0.5 min, 10–40% B from 0.5 to 1.5 min, 40–70% B from 1.5 to 5.5 min, 70–80% B from 5.5 to 8.5 min, 80–95% B from 8.5 to 8.7 min, held at 95% B from 8.7 to 11.5 min, 95–10% B from 11.5 to 11.7 min, and held at 10% B for the remaining 3.3 min. LC mobile phase was diverted to waste for the first four minutes and the last five minutes of the run. The eight steroids were monitored in positive ion mode using multiple-reaction monitoring (MRM); the transitions, declustering potentials, collision energies and collision cell exit potentials for the 8 steroids and deuterated internal standards are summarized in Table 2.

**Table 2:** Multiple-reaction monitoring (MRM) transitions, declustering potentials (DP), collision energies (CE) and collision cell exit potential (CXP) for the 8 steroids and deuterated internal standards.

Steroids	MRM transitions	DP	CE	CXP
Cortisol	36363/121	85	35	20
Cortisol-d4	367/121	85	32	20
Corticosterone	347/121	86	34	20
Corticosterone-d8	355/337	85	22	20
11-deoxycortisol	347/97	86	30	18
11-deoxycortisol-d2	349/97	86	42	16
DHEA	271/253	85	20	15
DHEA-d2	273/213	86	24	20
17β-Estradiol	255/159	85	28	5
17β-Estradiol-d4	259/160	85	28	10
Progesterone	315/97	86	30	16
Progesterone-d9	324/100	85	32	16
17-OH-Progesterone	331/97	85	31	15
17-OH-Progesterone-d8	339/100	85	32	18
Testosterone	289/97	85	34	10
Testosterone-d2	291/99	85	34	10

### Data and statistical analyses

Analyst software version 1.5 (AB SCIEX, Concord, ON) was employed for data acquisition, peak-area integration and quantitation of unknown serum or plasma samples. Calibration curves were derived in each analytical run, using calibrators in stripped serum matrix that passed through SPE with the sample batch. All experimental data were processed using Microsoft Excel 2007 software. The coefficient of variation (CV) was calculated as (standard deviation/mean)×100.

## Results

### Validation of the LC-MS/MS assay

**Sensitivity and Specificity:** Levels of endogenous steroids in stripped human serum were below the limit of quantitation (LOQ). The limit of quantitation (LOQ) for cortisol, corticosterone, 11-deoxycortisol, DHEA, E_2_, P_4_, 17-OH P and T_5_ in serum when analyzed simultaneously were 0.1, 0.05, 0.025, 0.1, 0.1, 0.1, 0.1 and 0.1 ng/mL respectively. These data were obtained based on the concentrations that produced a signal-to-noise (S/N) ratio of ≥10.

**Calibration curves:** Calibration curves for the eight steroids were obtained by linear regression analysis using internal standardization. Eight-point calibration curves were constructed and the linearity for each steroid was determined by plotting the peak area ratio of the analyte to I.S. versus the hormone concentration. The standard curves were generated from three independent runs and had correlation coefficients (R) between 0.9990 and 0.9999 (Table 3). Quantitation of the eight steroids in mammalian and avian samples was interpolated from the calibration curves. In general, the standard curve for each steroid used the LOQ as the lowest calibrator in the standard curve. This was modified only when expected values were much higher than the LOQ. For example, because the dominant glucocorticoid in birds is not cortisol, avian cortisol required a calibration curve ranging from 0.1–500 ng/mL, whereas mammalian cortiso was quantitated using a calibration curve ranging from 5–1000 ng/mL.

**Table 3:** Correlation coefficients (R) obtained from an eight-point calibration curve, recovery (in percent), intra-assay and inter-assay precision (coefficient of variation; CV) for each steroid.

	Cortisol	Corticosterone	11-deoxycortisol	DHEA	Estradiol	Progesterone	17-OH Progesterone	Testosterone
R (n = 3)	0.9993	0.9996	0.9999	0.9997	0.9995	0.9990	0.9993	0.9992
% Recovery (n = 3)	96	101	100	89	94	89	97	87
Intra-assay (n = 6)	5.00	6.24	7.80	9.69	7.89	1.11	8.24	5.70
Inter-assay (n = 4)	7.31	7.37	5.88	5.78	4.49	6.68	8.52	7.18

**Intra-Assay and Inter-Assay Precision:** Intra-assay precision was determined by analyzing 6 replicates of a quality control sample in a single LC-MS/MS run, while inter-assay precision was determined by running 4 replicates of a quality control sample on 4 different days. The percent coefficients of variation (CVs) for each steroid are shown in Table 3.

**Extraction Recovery:** Extraction recoveries of the 8 steroids following SPE were determined as follows: stripped human serum samples were spiked at the QC concentrations and submitted to extraction as described above. The dried samples were spiked with the I.S. in mobile phase. The mean peak area ratios of four processed samples were compared to un-extracted samples, where the analytes and I.S. were spiked after SPE. The results were expressed as percent recoveries (% recovery)  =  (extracted/unextracted)×100. The recoveries were high and consistent, ranging between 87 to 101%. The results are summarized in Table 3.

**Carry-over:** Carry-over was determined by running a blank solvent after the highest calibrator and by injecting a QC after an animal serum sample. No carry-over was detected.

### Optimization of sample preparation

**Established Sample Preparation Methods:** Steroids in serum are commonly extracted using liquid-liquid extraction or SPE [13], [14], [15]. We first extracted serum samples using ethyl acetate or diethyl ether. However, this procedure did not give clean extracts for LC-MS/MS analysis. That is, after eluates were dried and resuspended in 50% methanol, the extracts were cloudy or hazy (Table 4). SPE was then carried out for sample preparation using C18 columns, and water was used for washing to remove interferences in serum or plasma. However, when only a water wash was used, some extracts were still cloudy (Table 4). Thus, SPE optimization with multiple wash steps was necessary for the variety of serum and plasma samples tested.

**Table 4:** Serum and plasma samples were processed by liquid-liquid extraction (using ethyl acetate or diethyl ether), or by solid phase extraction (SPE) using water as the washing solvent, or by SPE using water and hexane as washing solvents (sequentially).

Species	Liquid-liquid extraction with ethyl acetate or diethyl ether	SPE with water wash only	SPE with water and hexane washes
Brown bear	cloudy	cloudy	clear
Camel	clear	clear	clear
Elephant	clear	clear	clear
Horse	clear	clear	clear
Moose	clear	not available	clear
Reindeer	clear	clear	clear
Tiger	cloudy	cloudy	clear
Albatross	cloudy	cloudy	clear
European starling	not available	cloudy	clear
House sparrow	not available	clear	clear
Song sparrow	not available	not available	clear
Zebra finch	not available	not available	clear

Observations were based on appearance of re-suspended sample by visual inspection comparing to a 50% methanol solution. Cloudy samples were not acceptable for LC-MS/MS injection.

**SPE optimization:** To optimize the SPE, we used samples from tigers and albatrosses, due to their high lipid content. Since hexane is effective for removing lipids from meat [16], this solvent was added as the second wash step, preceded by a water wash step. With a water wash and a hexane wash, clear extracts were obtained (Table 4), indicating that our SPE procedures were highly effective in removing interferences from samples with high lipid content. Note that ethyl acetate was used to elute the steroids, rather than 90% methanol (as in [17]). Ethyl acetate is preferable for high-throughput analysis, as it dries much more quickly, requiring less time for sample processing.

**Figure 1:**
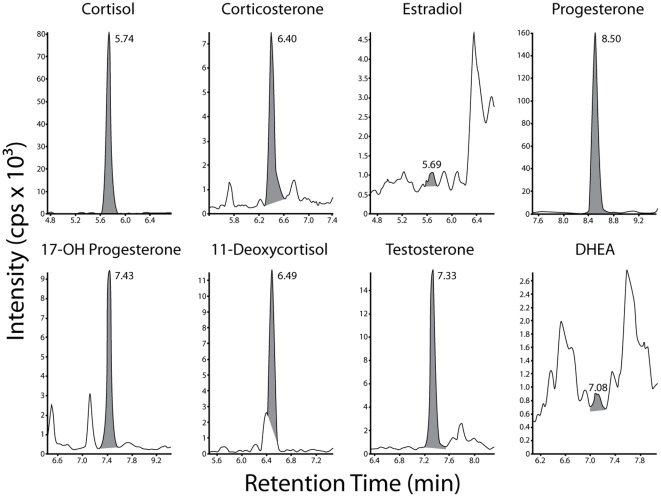
Ion chromatograms for a reindeer serum pool that included two males and two non-reproductive females. Cortisol, corticosterone, 11-deoxycortisol, progesterone, 17-OH progesterone and testosterone were present in this sample, but estradiol and DHEA were either not present or below the limit of quantitation. Transition details are presented in Table 2 and retention times (minutes) are indicated above the peak.

**Wildlife samples:** Using this SPE protocol, at least 6 endogenous steroids were identified in mammalian serum and avian plasma pools, using a single LC-MS/MS source sample (Figure 1; Table 5). With 100 µL of serum or plasma, 4–7 steroids were quantifiable (signal-to-noise (S/N) ratio ≥10) depending on the species and sample. Estradiol was not detected in mammalian pools that mixed male and female serum. However, estradiol was detected in some of the avian plasma pools. As expected, cortisol levels were higher than corticosterone levels in the mammalian samples, whereas the opposite was observed in the avian samples. Furthermore, as expected, testosterone and corticosterone levels were higher in breeding than non-breeding male song sparrows. Testosterone levels were also higher in breeding male than breeding female zebra finches.

**Table 5:** Steroid levels in mammalian sera or avian plasma (ng/mL).

Species	Cortisol	Corticosterone	11-deoxycortisol	DHEA	Estradiol	Progesterone	17-OH Progesterone	Testosterone
Brown bear	140.00	4.33	0.30	ND	ND	ND	0.48	2.64
Camel	27.10	4.92	0.08	ND	ND	ND	ND	0.18
Elephant	18.60	0.78	0.03	ND	ND	ND	0.25	6.43
Horse	64.70	1.33	0.06	0.26	ND	0.56	ND	0.15
Moose	24.10	2.22	0.09	ND	ND	1.01	ND	0.31
Reindeer	24.10	1.62	0.15	ND	ND	0.94	0.13	0.40
Tiger	442.00	18.4	0.38	ND	ND	0.19	0.19	3.57
Albatross; Breeding females	0.17	4.23	0.41	0.68	ND	0.37	ND	3.37
European starling; Breeding females	0.15	1.11	ND	0.45	0.19	0.13	ND	ND
House sparrow; Males and females	0.12	24.4	3.56	1.37	0.10	0.61	ND	0.10
Song sparrow; Breeding males	0.19	39.2	4.76	2.98	0.18	1.14	ND	3.64
Song sparrow; Non-breeding males	0.11	3.59	0.59	2.33	ND	0.33	ND	ND
Zebra finch; Breeding females	0.10	2.91	0.45	0.70	ND	0.32	ND	0.11
Zebra finch; Breeding males	0.12	4.68	0.54	0.66	ND	0.26	ND	0.51

For each species, serum or plasma was pooled from several subjects as detailed in the Methods. ND  =  not detected.

## Discussion

Using automated solid phase extraction (SPE) and liquid chromatography coupled to tandem mass-spectrometry (LC-MS/MS), we were able to validate an 8-steroid panel for comparative endocrine studies, similar to previous clinical work with human serum samples [9]. Slight reductions in sensitivity were primarily due to the low volume of the original sample (100 µl), the serum matrix used for the calibrators, and the SPE sample preparation of those calibrators. Comparable approaches for human serum have used initial serum volumes of 200–1000 µl, protein precipitation, and calibrators prepared inmethanol [9], [18], [19] or in methanol with bovine serum albumin [11].

The current method does not require protein precipitation, and uses hexane during the washing step to enhance lipid removal without losing steroid analytes. Following hexane, ethyl acetate worked well for elution and reduced drying time. In some previous studies using SPE for human serum, the washes used water only and the elution was done with methanol [11]. Diverse liquid-liquid extraction procedures, coupled to SPE, have also been developed for human serum [12], [19]. However, serum and plasma are often more lipid-rich in wildlife species. Although success has been achieved with protein precipitation and diethyl-ether extraction protocols for fish [10], the diversity of serum and plasma samples from wildlife in the current study were not uniformly suitable for LC-MS/MS following these established protocols.

Unlike the majority of our samples which combined male and female samples into the pool, our song sparrow and zebra finch avian pools were separated by sex and breeding season and could be compared with the broader literature. Direct comparisons of absolute steroid concentrations across different quantitation methods, including different kit manufacturers and different lot numbers, are rarely undertaken because of the large number of additional sample preparation/purification parameters that might differ. In addition, specific aspects of sample collection, including handling and social position, limit the usefulness of comparisons relative to our pooled samples. Nevertheless, results were generally congruent.

For example, Wingfield and Hahn (1994) measured concentrations of testosterone and corticosterone in male song sparrows using RIA following steroid separation [20]. Plasma pool concentrations in the current study were both higher and lower (Breeding male testosterone: 3.6 ng/mL versus ∼0.5±0.1 ng/mL; Breeding male corticosterone: 39 ng/mL versus ∼60±20 ng/mL; Non-breeding corticosterone: 4 ng/mL versus ∼20±10 ng/mL). Newman et al (2008) used direct RIAs of plasma and obtained breeding and non-breeding male song sparrow corticosterone concentrations similar to the Wingfield and Hahn values [21]. In the same study, DHEA concentrations were lower than the pools in the current study (Breeding male DHEA: 3 ng/mL versus ∼0.8±0.1 ng/mL; Non-breeding male DHEA: 2.3 ng/mL versus 0.75±0.1) [21]. For male zebra finches, concentrations were also both higher and lower in the published literature. For example, Taves et al. (2010) used SPE followed by RIA and found higher testosterone (Breeding male testosterone: 0.5 ng/mL versus ∼2±0.3 ng/mL) and higher DHEA (Breeding male DHEA: 0.7 ng/mL versus ∼3.4±0.7 ng/mL) but lower corticosterone (4.7 ng/mL versus ∼2.4±0.5 ng/mL) [22]. Charlier et al. (2010) used liquid-liquid extraction followed by RIA to yield a breeding male zebra finch testosterone concentration similar to Taves et al (2010) (2.5±0.7 ng/mL) [22], [23]. That study also quantified breeding male estradiol at ∼20±10 pg/mL. The current method, optimized for multiple steroids had an LOQ for estradiol well above that concentration (100 pg/mL), similar to Guo et al (2004) [18] and no estradiol was detected. As has been true for all of the diverse steroid quantitation approaches currently used for wildlife samples, the ability to match finer patterns of response across methods will require splitting individual samples for direct comparison, and was beyond the scope of the current study.

The limits of quantitation reported in the current study are not the limits of quantitation for individual steroids intrinsic to LC-MS/MS on the same instrument. For example, the limit of detection (as opposed to quantitation) for estradiol in human serum is as low as 2 pg/mL using similar equipment [24]. Thus, the advantages of obtaining multiple steroid quantitation from a single sample must always be traded-off against lost sensitivity for individual steroids when the expected concentration is low. Estradiol and other estrogens and metabolites are >clear examples where sensitivity can become a challenge [9], [18], [24].

Sample preparation for wildlife is challenging, because experimental designs are likely to include seasonal variation and population variation, and, therefore, variability in interfering compounds. The method reported here will allow diverse wildlife researchers to design experiments that maximize data acquisition from small, precious samples. In that context, confidence that a sample preparation method will not fail when a sample is unexpectedly more lipemic because of season, diet, or reproductive status, could be invaluable. Another advantage of this method is that there is no increase in sample analytic costs for obtaining multiple steroids, and overall costs are quite similar to ELISA or RIA approaches involving sample preparation. The potential applications of this method are therefore diverse. Previous wildlife research has not been able to effectively test hypotheses related to the co-variation of multiple steroids within individual samples.
